# Supplementary material for: Life expectancy and active life expectancy by disability status in older U.S. adults
Source: PLoS One. 2020 Sep 25;15(9):e0238890. doi: 10.1371/journal.pone.0238890 (PMC7518583; doi:10.1371/journal.pone.0238890)
Supplement: S1 Table — a: Standard error (SE) of estimated life expectancy at age x. b: SE of estimated active life expectancy at age x. c: SE of estimated life expectancy with activity limitation at age x. d: SE of estimate difference in total life expectancy (total) and active life expectancy (active). (PDF) [file pone.0238890.s001.pdf]

S1 Table. Standard Errors of Estimates in Table 2

Age (x)	Total sample			Initial disability status at age x							
				No Limitation			Activity Limitation			Difference[d]	
	e_x [a]	e_x^1 [b]	e_x^2 [c]	e_x	e_x^1	e_x^2	e_x	e_x^1	e_x^2	Total	Active
65	0.03	0.05	0.05	0.04	0.04	0.03	0.01	0.02	0.05	0.05	0.05
67	0.02	0.04	0.04	0.03	0.03	0.02	0.01	0.02	0.04	0.04	0.04
69	0.02	0.04	0.04	0.03	0.03	0.02	0.01	0.02	0.04	0.04	0.04
71	0.02	0.04	0.04	0.03	0.03	0.02	0.01	0.02	0.04	0.04	0.04
73	0.02	0.04	0.04	0.03	0.03	0.02	0.01	0.02	0.04	0.04	0.04
75	0.02	0.04	0.04	0.03	0.03	0.02	0.01	0.02	0.04	0.04	0.03
77	0.02	0.04	0.04	0.03	0.03	0.02	0.01	0.02	0.04	0.04	0.03
79	0.01	0.04	0.04	0.03	0.03	0.02	0.01	0.02	0.04	0.04	0.03
81	0.01	0.04	0.04	0.03	0.03	0.02	0.01	0.02	0.04	0.04	0.03
83	0.01	0.04	0.04	0.03	0.03	0.02	0.02	0.02	0.04	0.04	0.03
85	0.01	0.04	0.04	0.03	0.03	0.02	0.02	0.03	0.05	0.04	0.04
87	0.01	0.04	0.04	0.03	0.03	0.02	0.02	0.03	0.05	0.05	0.04
89	0.01	0.04	0.04	0.03	0.03	0.03	0.03	0.04	0.06	0.05	0.04
91	0.01	0.05	0.05	0.03	0.03	0.03	0.03	0.05	0.07	0.06	0.05
93	0.01	0.05	0.05	0.04	0.03	0.04	0.05	0.06	0.09	0.08	0.07
95	0.01	0.06	0.06	0.05	0.04	0.04	0.06	0.07	0.12	0.11	0.09

[a]: Standard error (SE) of estimated life expectancy at age x;
[b]: SE of estimated active life expectancy at age x;
[c]: SE of estimated life expectancy with activity limitation at age x;
[d]: SE of estimate difference in total life expectancy (total) and active life expectancy (active).
