# Supplementary material for: Life expectancy and active life expectancy by disability status in older U.S. adults
Source: PLoS One. 2020 Sep 25;15(9):e0238890. doi: 10.1371/journal.pone.0238890 (PMC7518583; doi:10.1371/journal.pone.0238890)
Supplement: S2 Table — a: Standard error (SE) of estimated life expectancy at age x. b: SE of estimated active life expectancy at age x. c: SE of estimated life expectancy with mild limitation at age x. d: SE of estimated life expectancy with disability at age x. (PDF) [file pone.0238890.s002.pdf]

S2 Table. Standard Errors of Estimates in Table 4

Age (x)	Total sample				Initial disability status at age x											
					No limitation				Mild limitation				Disability			
	e_x [a]	e_x^1 [b]	e_x^2 [c]	e_x^3 [d]	e_x	e_x^1	e_x^2	e_x^3	e_x	e_x^1	e_x^2	e_x^3	e_x	e_x^1	e_x^2	e_x^3
65	0.03	0.05	0.03	0.04	0.04	0.04	0.03	0.03	0.05	0.07	0.04	0.04	0.06	0.07	0.04	0.05
67	0.02	0.04	0.03	0.03	0.03	0.03	0.03	0.03	0.04	0.06	0.04	0.04	0.05	0.06	0.03	0.05
69	0.02	0.04	0.03	0.03	0.03	0.03	0.03	0.03	0.03	0.05	0.04	0.04	0.05	0.06	0.03	0.04
71	0.02	0.04	0.03	0.03	0.03	0.03	0.03	0.03	0.03	0.05	0.04	0.04	0.05	0.05	0.03	0.04
73	0.02	0.04	0.03	0.03	0.03	0.03	0.03	0.03	0.03	0.05	0.04	0.04	0.05	0.05	0.03	0.04
75	0.02	0.04	0.03	0.03	0.03	0.03	0.03	0.03	0.03	0.04	0.04	0.04	0.05	0.04	0.03	0.04
77	0.02	0.04	0.03	0.04	0.03	0.03	0.03	0.03	0.03	0.04	0.04	0.04	0.04	0.04	0.03	0.04
79	0.01	0.04	0.03	0.04	0.03	0.03	0.03	0.04	0.03	0.04	0.04	0.04	0.04	0.04	0.03	0.04
81	0.01	0.04	0.03	0.04	0.03	0.03	0.03	0.04	0.03	0.04	0.04	0.04	0.04	0.04	0.03	0.04
83	0.01	0.04	0.03	0.04	0.03	0.03	0.04	0.04	0.03	0.04	0.04	0.04	0.04	0.03	0.03	0.04
85	0.01	0.04	0.04	0.04	0.03	0.03	0.04	0.04	0.04	0.04	0.04	0.04	0.04	0.03	0.03	0.04
87	0.01	0.04	0.04	0.05	0.03	0.03	0.04	0.05	0.04	0.05	0.05	0.05	0.04	0.03	0.03	0.04
89	0.01	0.04	0.04	0.05	0.03	0.03	0.05	0.05	0.04	0.05	0.05	0.05	0.04	0.03	0.03	0.05
91	0.01	0.05	0.05	0.06	0.03	0.03	0.05	0.06	0.05	0.05	0.06	0.06	0.04	0.03	0.03	0.05
93	0.01	0.05	0.06	0.07	0.04	0.03	0.06	0.08	0.06	0.06	0.08	0.08	0.05	0.02	0.03	0.06
95	0.01	0.06	0.06	0.08	0.05	0.04	0.07	0.10	0.07	0.07	0.09	0.09	0.05	0.02	0.03	0.06

[a]: Standard error (SE) of estimated life expectancy at age x;
[b]: SE of estimated active life expectancy at age x;
[c]: SE of estimated life expectancy with mild limitation at age x;
[d]: SE of estimated life expectancy with disability at age x.
