# Supplementary material for: Life expectancy and active life expectancy by disability status in older U.S. adults
Source: PLoS One. 2020 Sep 25;15(9):e0238890. doi: 10.1371/journal.pone.0238890 (PMC7518583; doi:10.1371/journal.pone.0238890)
Supplement: Attachment — Submitted filename: Responses to editor and reviewers.docx [file pone.0238890.s003.docx]

PONE-D-20-17729
Life expectancy and active life expectancy by disability status in older U.S. adults: results from the Medicare Health Outcome Survey (HOS)
PLOS ONE

Editor comments and journal Requirements:

When submitting your revision, we need you to address these additional requirements.

1. Please ensure that your manuscript meets PLOS ONE's style requirements, including those for file naming. The PLOS ONE style templates can be found at

<https://journals.plos.org/plosone/s/file?id=wjVg/PLOSOne_formatting_sample_main_body.pdf> and <https://journals.plos.org/plosone/s/file?id=ba62/PLOSOne_formatting_sample_title_authors_affiliations.pdf>

**Response:** We have formatted the manuscript to meet PLOS ONE’s requirements

2. In ethics statement in the manuscript and in the online submission form, please provide additional information about the patient records used in your retrospective study. Specifically, please ensure that you have discussed whether all data were fully anonymized before you accessed them and/or whether the IRB or ethics committee waived the requirement for informed consent. If patients provided informed written consent to have data from their medical records used in research, please include this information.

**Response:** This study is a secondary data analysis using the Limited Data Set (LDS) of the HOS from the CMS. The HOS LDS files retain some protected beneficiary-level health information, such as date of birth, gender, race/ethnicity, and county of residence; however, specific direct person identifiers (i.e., name, address, HIC number, and SSN) are not included in the LDS files, as outlined in the Health Insurance Portability and Accountability Act (HIPAA Privacy Rule) (<https://www.hosonline.org/en/data-dissemination/research-data-files/>).

3. Please consider including your recent study (Jia, Haomiao, et al. "Quality-adjusted life years (QALYs) associated with limitations in activities of daily living (ADL) in a large longitudinal sample of the US community-dwelling older population." Disability and health journal 12.4 (2019): 699-705.) in the discussion, to provide a more complete review of the topic.

**Response:**  We added the following to the Discussions:

Recently, Jia et al. (2019) examined the impact of ADL limitations on quality-adjusted life years (QALYs), a multi-dimensional summary measure of health that includes both morbidity and mortality, in the same HOS data [31]. The analysis demonstrated the incremental decrease in QALYs with worsening ADL limitations and illustrated that ADL statuses were more predictive of health-related quality of life (HRQOL) and mortality than chronic conditions. However, the analysis was based on the assumption that participants will remain in a given ADL status until death. Therefore, these estimates do not provide the exact QALY loss due to ADL, but, rather, the difference in QALYs between respondents with any ADL limitations throughout remaining lifetime and respondents without an ADL limitation throughout remaining lifetime.

4. We note that you have indicated that data from this study are available upon request. PLOS only allows data to be available upon request if there are legal or ethical restrictions on sharing data publicly. For information on unacceptable data access restrictions, please see <http://journals.plos.org/plosone/s/data-availability#loc-unacceptable-data-access-restrictions>.

In your revised cover letter, please address the following prompts:

a) If there are ethical or legal restrictions on sharing a de-identified data set, please explain them in detail (e.g., data contain potentially identifying or sensitive patient information) and who has imposed them (e.g., an ethics committee). Please also provide contact information for a data access committee, ethics committee, or other institutional body to which data requests may be sent.

b) If there are no restrictions, please upload the minimal anonymized data set necessary to replicate your study findings as either Supporting Information files or to a stable, public repository and provide us with the relevant URLs, DOIs, or accession numbers. Please see <http://www.bmj.com/content/340/bmj.c181.long> for guidelines on how to de-identify and prepare clinical data for publication. For a list of acceptable repositories, please see <http://journals.plos.org/plosone/s/data-availability#loc-recommended-repositories>.

We will update your Data Availability statement on your behalf to reflect the information you provide.

**Response:** The data used in this study were from a LDS from CMS. The dataset contains potentially identifying or sensitive patient information (e.g., participants’ zip code, date of birth, date of death, etc.). A signed Data Use Agreement (DUA) with CMS is required to obtain LDS data files. The data file was obtained through a DUA between the U.S. Center for Disease Control and Prevention (CDC) and the CMS. In this DUA, the corresponding author (HJ) is one of custodians of the data file. The authors do not have the authority share the data publically.

Reviewer comments:

Reviewer's Responses to Questions

**Comments to the Author**

1. Is the manuscript technically sound, and do the data support the conclusions?

The manuscript must describe a technically sound piece of scientific research with data that supports the conclusions. Experiments must have been conducted rigorously, with appropriate controls, replication, and sample sizes. The conclusions must be drawn appropriately based on the data presented.

Reviewer #1: Yes

Reviewer #2: Yes

2. Has the statistical analysis been performed appropriately and rigorously?

Reviewer #1: I Don't Know

Reviewer #2: I Don't Know

3. Have the authors made all data underlying the findings in their manuscript fully available?

The [PLOS Data policy](https://urldefense.proofpoint.com/v2/url?u=http-3A__www.plosone.org_static_policies.action-23sharing&d=DwMGaQ&c=G2MiLlal7SXE3PeSnG8W6_JBU6FcdVjSsBSbw6gcR0U&r=stxhwZX7w8bPzpef8t2Fuo53abx-784IIQ4JTvcQG6s&m=CMT0wsEb0x5DDTCWAe0sMc9JpciM2c4jJxsdywFeUvM&s=A7PnsjVJvPuXIg7IdQ9SmPYV369rzNT7bR9dhISgBks&e=) requires authors to make all data underlying the findings described in their manuscript fully available without restriction, with rare exception (please refer to the Data Availability Statement in the manuscript PDF file). The data should be provided as part of the manuscript or its supporting information, or deposited to a public repository. For example, in addition to summary statistics, the data points behind means, medians and variance measures should be available. If there are restrictions on publicly sharing data—e.g. participant privacy or use of data from a third party—those must be specified.

Reviewer #1: Yes

Reviewer #2: Yes

4. Is the manuscript presented in an intelligible fashion and written in standard English?

PLOS ONE does not copyedit accepted manuscripts, so the language in submitted articles must be clear, correct, and unambiguous. Any typographical or grammatical errors should be corrected at revision, so please note any specific errors here.

Reviewer #1: Yes

Reviewer #2: Yes

5. Review Comments to the Author

Please use the space provided to explain your answers to the questions above. You may also include additional comments for the author, including concerns about dual publication, research ethics, or publication ethics. (Please upload your review as an attachment if it exceeds 20,000 characters)

Reviewer #1: I'm not really picking up on any existential flaws, although a statistician might be better at critically probing the modeling approach. It's basically a secondary analysis of a large data set that shows health status relates to both active and disabled lifespans, which seems unsurprising. It's certainly well-written and I suspect there are some in the public health field who might find this of interest.

Reviewer #2: Upon reviewing the manuscript, I felt that it was well drafted and scientifically coherent. It is a novel topic as it explores the unique question of how many years of life expectancy years are spent active and independent vs in disability requiring assistance. One recommendation I have is for the author is elucidate upon the chronic disorders that the participants had and how those directly or indirectly affected their disability status. As the study aims to create a database for healthcare providers, a sound understanding of the various chronic conditions like diabetes, hypertension, obesity, etc of the participants and how those posed as a disability in their lives would help healthcare providers predict life expectancy and active life expectancy for those who have those specific conditions. Special mention could also be made of disorders that cause cognitive decline like Alzheimer's Disease and dementia and how those too can impact the disability status and life expectancy of a person. This additional knowledge can help health care providers design specific treatment plans catered to the specific disorders and help them increase the life expectancy and/or the active life expectancy of their geriatric patients.

**Response:** We agree with the reviewer that chronic disorders may directly or indirectly affect the participant’s disability status. Other investigators and organizations have examined the intersection between chronic diseases and disability (Gulley et al, 2018). For example, the Institute of Medicine’s *Living well with chronic illness: A call for public health action* describes a distribution of population level subgroups that spans from healthy, to at risk, to chronic illness without impairment, to impairment, to functional limitations, and to disability (IOM 2012). We previously published a manuscript the impact of ADLs on quality-adjusted life years (QALYs) (Jia et al. 2019). The analysis illustrated that ADL statuses were more predictive of health-related quality of life (HRQOL) and mortality than chronic conditions. We have added a paragraph on the findings of this manuscript, and how the current investigation adds to the literature, but a full discussion of the impact of chronic diseases is beyond the scope of this manuscript.

We also agree with the reviewer that disorders associated with cognitive decline impact the disability status and life expectancy of a person. Unfortunately, the Medicare HOS Cohort 15 did not include an item about memory problems. However, the more recent versions of the HOS (2018, 2019, and 2020) include the following item: In the past month, how often did memory problems interfere with your daily activities? This would be an interesting item to examine in a future analysis. Of note, CMS has not announced the date of dissemination of HOS data from 2018 or later.

References:

- Jia H, Lubetkin EI, DeMichele K, Stark DS, Zack MM, Thompson WW. [Quality-adjusted life years (QALYs) associated with limitations in activities of daily living (ADL) in a large longitudinal sample of the U.S. community-dwelling older population.](https://www.ncbi.nlm.nih.gov/pubmed/31167742) *Disabil Health J*. 2019; 12(4):699-705.

# Gulley SP, Rasch EK, Bethell CD, Carle AC, Druss BG, Houtrow AJ, Reichard AJ, Chan L. At the intersection of chronic disease, disability and health services research: A scoping literature review. [*Disabil Health J*. 2018; 11(2): 192–203.](https://www.ncbi.nlm.nih.gov/entrez/eutils/elink.fcgi?dbfrom=pubmed&retmode=ref&cmd=prlinks&id=29396271)

- Institute of Medicine. Living well with chronic illness: A call for public health action. Washington, DC: The National Academies Press; 2012.

6. PLOS authors have the option to publish the peer review history of their article ([what does this mean?](https://urldefense.proofpoint.com/v2/url?u=https-3A__journals.plos.org_plosone_s_editorial-2Dand-2Dpeer-2Dreview-2Dprocess-23loc-2Dpeer-2Dreview-2Dhistory&d=DwMGaQ&c=G2MiLlal7SXE3PeSnG8W6_JBU6FcdVjSsBSbw6gcR0U&r=stxhwZX7w8bPzpef8t2Fuo53abx-784IIQ4JTvcQG6s&m=CMT0wsEb0x5DDTCWAe0sMc9JpciM2c4jJxsdywFeUvM&s=xmfNBA8ePGbtGD-klTMarnVR77o_dXnXiRNxjk_q0ek&e=)). If published, this will include your full peer review and any attached files.


If you choose “no”, your identity will remain anonymous but your review may still be made public.


**Do you want your identity to be public for this peer review?** For information about this choice, including consent withdrawal, please see our [Privacy Policy](https://urldefense.proofpoint.com/v2/url?u=https-3A__www.plos.org_privacy-2Dpolicy&d=DwMGaQ&c=G2MiLlal7SXE3PeSnG8W6_JBU6FcdVjSsBSbw6gcR0U&r=stxhwZX7w8bPzpef8t2Fuo53abx-784IIQ4JTvcQG6s&m=CMT0wsEb0x5DDTCWAe0sMc9JpciM2c4jJxsdywFeUvM&s=2j_26VRr0QKSdSpGbMlfnQLFCqe2rzTGdaY6NiRboGs&e=).

Reviewer #1: No

Reviewer #2: No

[NOTE: If reviewer comments were submitted as an attachment file, they will be attached to this email and accessible via the submission site. Please log into your account, locate the manuscript record, and check for the action link "View Attachments". If this link does not appear, there are no attachment files.]

While revising your submission, please upload your figure files to the Preflight Analysis and Conversion Engine (PACE) digital diagnostic tool, [https://pacev2.apexcovantage.com/](https://urldefense.proofpoint.com/v2/url?u=https-3A__pacev2.apexcovantage.com_&d=DwMGaQ&c=G2MiLlal7SXE3PeSnG8W6_JBU6FcdVjSsBSbw6gcR0U&r=stxhwZX7w8bPzpef8t2Fuo53abx-784IIQ4JTvcQG6s&m=CMT0wsEb0x5DDTCWAe0sMc9JpciM2c4jJxsdywFeUvM&s=2poGOFnGPdWpbywJz0Fh42awy49QSHdRhxOV1vMv6gQ&e=). PACE helps ensure that figures meet PLOS requirements. To use PACE, you must first register as a user. Registration is free. Then, login and navigate to the UPLOAD tab, where you will find detailed instructions on how to use the tool. If you encounter any issues or have any questions when using PACE, please email PLOS at [figures@plos.org](mailto:figures@plos.org). Please note that Supporting Information files do not need this step.

*In compliance with data protection regulations, you may request that we remove your personal registration details at any time.* [*(Remove my information/details)*](https://urldefense.proofpoint.com/v2/url?u=https-3A__www.editorialmanager.com_pone_login.asp-3Fa-3Dr&d=DwMGaQ&c=G2MiLlal7SXE3PeSnG8W6_JBU6FcdVjSsBSbw6gcR0U&r=stxhwZX7w8bPzpef8t2Fuo53abx-784IIQ4JTvcQG6s&m=CMT0wsEb0x5DDTCWAe0sMc9JpciM2c4jJxsdywFeUvM&s=dAyDQnGigxxOB8Gf1y4boRBgv2UDiws1B8cJXPHu9ZA&e=)*. Please contact the publication office if you have any questions.*
